# Effect of Secondary Carbon Nanofillers on the Electrical, Thermal, and Mechanical Properties of Conductive Hybrid Composites Based on Epoxy Resin and Graphite

**DOI:** 10.3390/ma14154169

**Published:** 2021-07-27

**Authors:** Marcel Zambrzycki, Krystian Sokolowski, Maciej Gubernat, Aneta Fraczek-Szczypta

**Affiliations:** Department of Biomaterials and Composites, Faculty of Materials Science and Ceramics, AGH University of Science and Technology, Mickiewicza 30 Av., 30-059 Cracow, Poland; zambrzycki@agh.edu.pl (M.Z.); ksokolow@agh.edu.pl (K.S.); maciej.gubernat@agh.edu.pl (M.G.)

**Keywords:** conductive polymer composite, bipolar plates, graphene, carbon black, graphite, epoxy, electrical conductivity

## Abstract

In this work, we present a comparative study of the impact of secondary carbon nanofillers on the electrical and thermal conductivity, thermal stability, and mechanical properties of hybrid conductive polymer composites (CPC) based on high loadings of synthetic graphite and epoxy resin. Two different carbon nanofillers were chosen for the investigation—low-cost multi-layered graphene nanoplatelets (GN) and carbon black (CB), which were aimed at improving the overall performance of composites. The samples were obtained by a simple, inexpensive, and effective compression molding technique, and were investigated by the means of, i.a., scanning electron microscopy, Raman spectroscopy, electrical conductivity measurements, laser flash analysis, and thermogravimetry. The tests performed revealed that, due to the exceptional electronic transport properties of GN, its relatively low specific surface area, good aspect ratio, and nanometric sizes of particles, a notable improvement in the overall characteristics of the composites (best results for 4 wt % of GN; *σ* = 266.7 S cm^−1^; *λ* = 40.6 W mK^−1^; fl. strength = 40.1 MPa). In turn, the addition of CB resulted in a limited improvement in mechanical properties, and a deterioration in electrical and thermal properties, mainly due to the too high specific surface area of this nanofiller. The results obtained were compared with US Department of Energy recommendations regarding properties of materials for bipolar plates in fuel cells. As shown, the materials developed significantly exceed the recommended values of the majority of the most important parameters, indicating high potential application of the composites obtained.

## 1. Introduction

Conductive polymer composites (CPC) constitute a dynamically developing class of modern materials, representing a promising alternative for many applications, e.g., bipolar plates for low- and medium-temperature fuel cells and batteries, i.e., polymer exchange membrane fuel cells (PEMFC), alkaline fuel cells, and vanadium redox battery (VRB) [1,2]. The most important feature of CPC is its electrical conductivity, usually achieved using graphite, carbon fibers or carbon black as the primary conductive filler and smaller amounts of secondary additives, e.g., carbon nanotubes [3,4,5]. The purpose of combining polymers with graphite or other conductive fillers in CPC is to achieve a percolation threshold, enabling the formation of uninterrupted conduction paths in the material. Additionally, a secondary conductive filler can be added which usually aims at providing a better physical contact between the adjacent particles of the primary filler and reducing of the contact resistance between the neighboring grains, shortening of the conduction paths and an enlarging effective cross-sectional area of the conductor. The effectiveness of the modification depends on the type of additive, above all its electronic structure, size of nanoparticles, shape and aspect ratio, its degree of dispersion, its specific surface area, interaction with the polymer matrix, and purity [6,7,8]. Carbon fibers and carbon nanotubes, due to their high aspect ratio, facilitate the achievement of appropriate connections and conductivity at lower proportions than in the case of low aspect ratio particles, as has been confirmed in numerous scientific studies [6,9,10]. Because of their high aspect ratio, graphene flakes should also show a high potential in this area; however, the number of scientific works on their application as a secondary modifier for CPC composites in bipolar plates is still limited, which highlights the need to expand knowledge in this area [11].

Fuel cell operation is based on the process of catalytic redox reactions, which take place on the catalytic bed, where water is usually the only by-product. This makes this type of device beneficial and promising from the environmental point of view [12]. The theoretical energy efficiency of PEMFC cells reaches 83%, but in practice it does not usually exceed the level of 40–65%. In order to increase the voltage (about 1 V per cell for PEMFC) and current generated, individual cells are combined into stacks using interconnectors, also known as bipolar plates. They are important components which ensure a number of functions of fuel cells, namely, bringing the reaction gases into the vicinity of the bed, physically connecting the whole cell stack, and enabling the flow of electric current from electrodes to the current collectors. The recommendations for the material properties of bipolar plates are given by US Department of Energy (DOE) and are as follows: electrical conductivity (>100 S cm^−1^), thermal conductivity (>10 W mK^−1^), surface resistance ASR (<0.01 Ω cm^2^), H_2_ permeation coefficient (1.3 × 10^−14^), flexural strength (>25 MPa), corrosion resistance (<1 μA cm^−2^; from voltametric methods), low weight (0.4 kg kWnet^−1^), and low cost (3 $ kWnet^−1^) [13,14,15,16,17,18]. The modifications of interconnectors are one of the most important approaches for improvements in the efficiency of fuel cells. The most important issues regarding this topic are attempts to reduce the weight of the stack—increasing its energy density, preventing the corrosion of metallic plates and also preventing brittle failure of plates made from other materials, such as pure graphite [19,20]. The forementioned problems are all the more important because bipolar plates constitute approx. 80% of the whole cell mass, and are responsible for approx. 30–60% of their total production costs [18]. Composite bipolar plates from CPC are fabricated on the basis of inexpensive and widely available major components, most commonly graphite and polymer matrix, which have the potential to meet price, electrical, mechanical, corrosion resistance, and low-density requirements. An important additional advantage is simple and cheap production technology of CPC production through compression molding.

The aim of this work was to develop light, highly conductive CPCs based on epoxy resin, graphite, and graphene flakes with potential application as materials for bipolar plates for PEMFC and to investigate the impact of the addition of graphene nanoplatelets on the electrical, thermal, and mechanical properties of the hybrid composites produced. The results obtained were compared with the analogous composites based on carbon black, which is the material most commonly used as the additional secondary conductive filler for CPC. Particular attention was paid to what influence the specific surface area and aspect ratio of the nanoparticles used had on the enhancement of properties of composites. As result of this study, we obtained the composites meeting most of the DOE requirements for bipolar plates, significantly exceeding some of these.

## 2. Materials and Methods

### 2.1. Materials

Synthetic graphite (SG) provided by SGL Carbon SE (Wiesbaden, Germany) was used as the primary conductive filler. Multi-layered graphene nanoplatelets (GN; AO3 grade) and carbon black (CB) were used as the secondary nanofillers—both purchased from Graphene Laboratories Inc (Ronkonkoma, NY, USA). General characteristics of the above have been discussed in the Characterization of raw materials section, and the most important parameters were presented in Table 1. Low-viscosity, bisphenol-A based epoxy resin LH288 (*η* ~ 500–900 mPa s in 25 °C; epoxy index ~ 0.54 mol kg^−1^) and H282 crosslinking agent based on N,N’-bis (2-aminoethyl)-ethylenediamine were purchased from Havel Composites PL (Cieszyn, Poland).

### 2.2. Preparation of Nanocomposites

Prior to the molding, the primary and secondary fillers were mixed in the ball mill for 60 min, and after that they were added to the epoxy resin and mixed for another 5 min. Subsequently, the curing agent was added (epoxy-hardener wt. ratio—100:23) and the mixing process was repeated. The obtained mixtures were transferred into rectangular stainless steel molds and subjected to hot uniaxial pressing under the following conditions: *p* = 20 MPa, *T* = 85 °C, *t* = 20 min. After holding the samples for the aforementioned duration, the moldings were allowed to cool down under the applied pressure and demolded at room temperature. In order to exclude the influence of the skin-effect related to the presence of a polymer-rich layer at the surface of specimens, their top layer was polished using microgrit P400 sandpaper. The basic composition of materials was established during our previous experiments and contained 15 wt % of epoxy resin as binder and 85 wt % of SG as primary conductive filler. Such composition ensured good mechanical properties of composite and did not caused outflow of the resin from the mold during pressing. The addition of secondary nanofillers was performed at the expense of graphite content, so that the total filler content remained constant at 85 wt %. In the case of GN, contents of from 0 to 6 wt % were tested, while in the case of CB, due to its relatively lower cost, the range of evaluated contents was extended to 8 wt %. Following this procedure, one composite and eight types of nanocomposites were obtained:

- EP/SG—A composite based on epoxy resin and 85 wt % of graphite, ref. sample.
- 1% GN—A nanocomposite based on epoxy resin and graphite with 1 wt % of GN.
- 2% GN—A nanocomposite based on epoxy resin and graphite with 2 wt % of GN.
- 4% GN—A nanocomposite based on epoxy resin and graphite with 4 wt % of GN.
- 6% GN—A nanocomposite based on epoxy resin and graphite with 6 wt % of GN.
- 2% CB—A nanocomposite based on epoxy resin and graphite with 2 wt % of CB.
- 4% CB—A nanocomposite based on epoxy resin and graphite with 4 wt % of CB.
- 6% CB—A nanocomposite based on epoxy resin and graphite with 6 wt % of CB.
- 8% CB—A nanocomposite based on epoxy resin and graphite with 8 wt % of CB.

### 2.3. Methods

The observations of morphology and microstructure of materials in the magnification range of 350 to 40,000× were taken using FEI Nova NanoSEM 200 scanning electron microscope (SEM; Eindhoven, Netherlands) equipped with a low vacuum detector (LVD). The applied acceleration voltage was 10 kV. The obtained images were analyzed using ImageJ v1.53e software. Raman measurements were taken using a Horiba LabRAM HR spectrometer (Longjumeau, France) coupled with a digital camera and a 532 nm laser excitation source. In order to ensure statistical validity, three separate measurements were carried out for each sample. Spectra were analyzed using Fityk v0.8.0 software [21]. Deconvolution of the first order spectrum was performed using the Pseudo Voight function. Measurements of electrical conductivity were made using a TMT-2 K double bridge. In order to provide proper electrical contact, the sandpapered edges of rectangular samples were covered with SPI conductive silver paint. Thermal conductivity of composites was determined by the laser flash analysis (LFA) method using a Netzsch LFA 427 (Selb, Germany) working with an InSb sensor. The measurements were carried out at 25 °C under the 150 mL min^−1^ Ar protective flow. The samples had the form of plates with dimensions of 10 × 10 × 2 mm^3^. Three independent measurements were taken for each sample. For the purpose of calculation of thermal conductivity, we assumed the average value of specific heat capacity of composites to be equal 0.874 J g^−1^ K^−1^ as calculated from the rule of mixtures—c_p-COMP_ = 0.15c_p-EP_ + 0.85c_p-SG_ [22,23]. Thermogravimetry analysis (TG) was made using Netzsch STA 449 F3 Jupiter (Selb, Germany). The samples were heated under the 99.999% nitrogen protective atmosphere flow of 50 mL min^−1^ up to 550 °C. The heating rate was 10 K min^−1^. Three-point bending tests according to the ASTM D790-03 standard were made using a Zwick 1425 universal testing machine (Ulm, Germany). Hardness measurements according to ASTM D2240 standard were made using a Shore durometer LX-AD.

## 3. Results and Discussion

### 3.1. Characterization of Raw Materials

**Figure 1:**
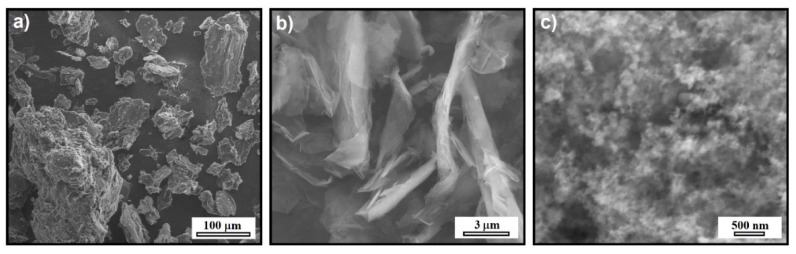
SEM images of the carbon fillers (**a**) synthetic graphite, (**b**) graphene nanoplatelets, and (**c**) carbon black nanoparticles.

The carbon filler powders were examined by SEM for grain shape and size prior to the molding of the composite plates. Figure 1 shows SEM images of three types of commercial carbon powders: graphite, graphene, and carbon black particles used as multi-objective fillers to improve the general properties of epoxy-based composites.

The images show that the carbon powders used as fillers in composites differed in shape, size, and distribution of the grains. The graphite powder shown in Figure 1a had an irregular, flake-like grain shape with a corrugated surface. The SG particle size was in the range of 10–200 µm, and in the case of graphene powder (Figure 1b), a large compaction of the grains in the form of regular plates with a smooth surface was observed. The lateral sizes of plates were from 1.5 to 10 µm. In turn, as shown in Figure 1c, the carbon black powder consisted of spherical particles with an average size of 30 nm. Table 1 presents a summary of the information regarding the carbon fillers derived from SEM analysis and other data provided by suppliers.

As shown in Table 1, the carbon fillers had various values of specific surface area due to differences in their size, shape, and pore structure. The greatest SSA, i.e., 254 m^2^ g^−1^ was noted for CB powder which contains homogeneous spheroidal-like particles in nanometre-sizes and with a well-developed surface. Graphene nanoplatelets, due to their multi-layered character, had a relatively low value of SSA ~ 80 m^2^ g^−1^. The smallest SSA = 1.94 m^2^ g^−1^ resulting from the largest sizes of irregular platelets was noted for graphite. It is worth noting that, in the case of highly loaded with fillers CPCs, the SSA value of additional secondary filler strictly determines its amount that can be loaded into the matrix while maintaining good wetting of particles with the binder, ensuring proper microstructural integrity of the composite. Thus, it determines the magnitude of the changes in the values of properties of the primary microfiller-based composites containing additional nanofiller particles [10].

**Figure 2:**
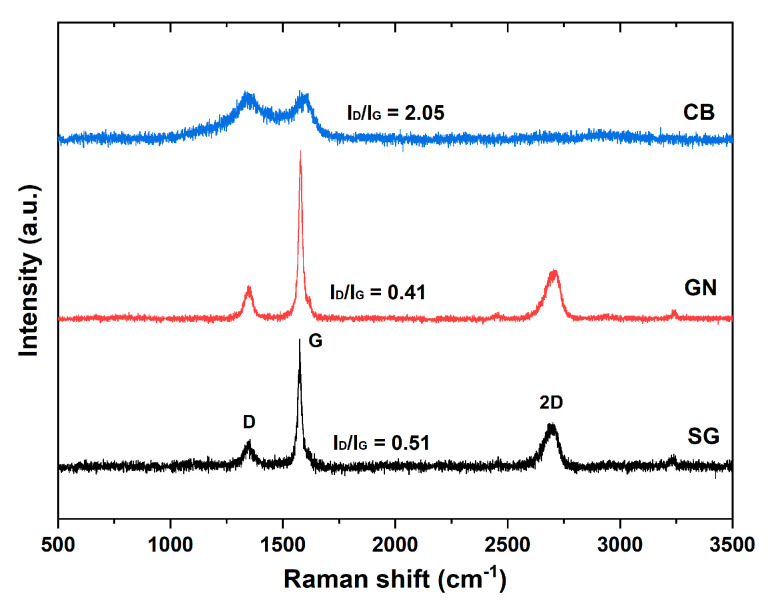
Raman spectra of the carbon fillers used.

All carbon fillers were also characterized in terms of structural ordering using Raman spectroscopy. Figure 2 compares the Raman spectra for CB, GN, and SG samples measured with a 532 nm laser.

As shown in Figure 2, the spectra of carbon fillers exhibited various shapes, intensities, and Raman shifts of the peaks. The graphene and graphite spectra were similar, while the CB spectrum differed significantly from them. Firstly, three peaks at ~1355 cm^−1^, 1575 cm^−1^, and 2700 cm^−1^, are visible in the SG and GN spectra. These are peaks known as first-order D band (disordered carbon) and G band (graphitic carbon) and the second order 2D band [24]. The G peak originates from the stretching vibration of sp^2^ (C-C) carbon atoms, whereas the D band is attributed to the topological disorder of the carbon structure, the presence of sp^3^ and boundary defects, etc. In turn, the 2D peak is related to the ordering along the c-axis and can be used to monitor the number of graphene layers [25,26].

The shape and intensity of the G and D peaks in SG and GN were similar—in both, the G peak is sharp and narrow, while the D peak has low intensity. The sharpening of the G peak, decrease of the intensity of D peak and the appearance of the 2D band are characteristic features of spectra indicating the three-dimensional order of sp^2^ carbons [26]. The 2D peak has a similar shape and intensity in both SG and GN samples, which results mainly from the multi-layered structure of graphene. In the case of CB, the D and G peaks are centered at 1348 and 1592 cm^−1^ and are significantly widened due to the presence of structural disorder.

The degree of ordering of carbons was evaluated by analysis of the ratio of the integral intensities of the D and G Raman bands (I_D_/I_G_). Its values for CB, SG, and GN were 2.05, 0.51, and 0.41, respectively. This indicates that carbon black had a strongly disordered structure, with a large number of sp^3^ bonds and defects, while the structures of SG and GN were highly ordered and crystalline. Here it should be emphasized that the degree of structural ordering and sp^2^ carbon percentage is strictly related to the electrical conductivity of carbon materials and it can be expected that conductivity of fillers should be in some way proportional to the reciprocal of the I_D_/I_G_ ratio [27].

### 3.2. Microstructure of Composites

**Figure 3:**
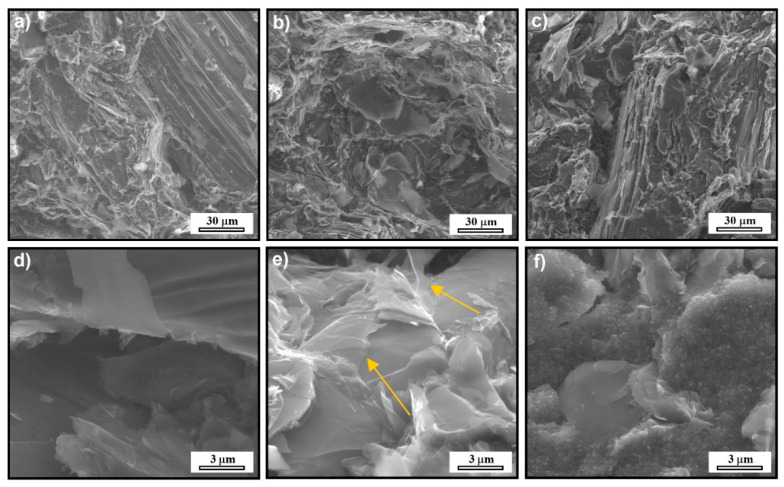
SEM images of EP/SG composite without nanofillers (**a**,**d**); with graphene nanoplatelets (**b**,**e**); with carbon black (**c**,**f**).

The morphology and microstructure of EP/SG composites modified with GN and CB were investigated using SEM. The fracture surfaces of composites also provide information about the failure mechanism and influence of GN and CB on the failure process. All types of composites showed a similar, rough fracture surface, not resembling the smooth fracture characteristic of the epoxy resin, which is the result of the presence of the modifying phase in the form of graphite. The increased surface roughness implies that the path of the crack tip was distorted because of the graphite phase, making crack propagation more difficult [28,29]. Low magnification images for composites with GN and CB show (Figure 3b,c) no significant differences in relation to the reference sample (Figure 3a). The higher magnification SEM micrographs in Figure 3e and f show that the presence of GN and CB affected the microstructure of the fracture surface, especially for composites containing CB. In the case of composites with graphene nanoplates (Figure 3b), the morphology of the fracture surface of the sample is similar to the reference sample and all phases are well wetted/bonded by the polymer. The SEM image of these samples shows the presence of graphene flakes covering the graphite grains (Figure 3e, arrows). The distribution of GN in the composite seems to be homogeneous, which results in an increase in the mechanical and electrical properties of this type of nanocomposites. In the case of CB modified nanocomposites (Figure 3f), the fracture surface is very rough, the modifying phase is very clearly visible in the form of spherical particles with a size adequate to the CB particles shown in Figure 1c. This phase fills the entire volume of the resin very strongly, possibly leading to a problem with wetting/binding it with the resin, especially at weight percentages above 2%. The reason for this is that the large surface area of the CB (SSA = 254 m^2^ g^−1^) causes the resin to be quickly saturated by the excessive surface of the particles even at small weight fractions, contributing to the problem of wetting at higher concentrations of nanoadditives.

### 3.3. Electrical Conductivity

**Figure 4:**
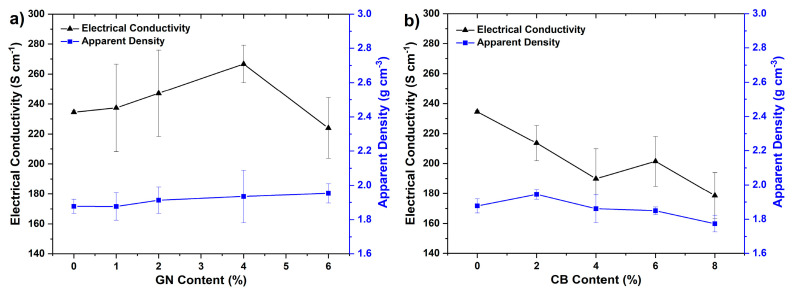
Electrical conductivity and apparent density of composites depending on the content of secondary nanofillers: (**a**) graphene nanoplatelets, (**b**) carbon black.

The electrical conductivity and apparent density of composites depending on the content of the added secondary nanofillers are shown in Figure 4. For composites with graphene nanoplatelets, the electrical conductivity increased with the rise in filler content up to 4 wt % (*σ* = 266.7 S cm^−1^), after which point it stopped increasing. The apparent density of these composites was in the range of 1.88–1.95 g cm^−3^ and also increased along with the GN content due to the denser packaging of the filler grains resulting directly from its more complex gralnulometric composition [4,30]. The observed enhancement of the electrical conductivity was particularly due to the forementioned denser packaging of fillers which resulted in improved physical contact between the single grains of graphite, prolonged the non-interrupted path of conduction and enlarged the effective cross-sectional area of the conductor (enhanced percolation network) [31]. Obviously, the excellent conductivity of graphene was the primary factor which enabled improvements in the conductivity of composites. With regard to carbon black, its addition resulted in a gradual decrease in the conductivity of composites in line with the growing content of CB. The most likely reasons for the observed decline were the relatively low conductivity of CB as compared to SG, resulting from the strongly disordered structure of CB affecting the charge transport in it, as well as the deterioration in overall microstructural binding and integrity of composites [4,32]. This second factor can be inferred from the dependence of the apparent density of samples on the content of CB, which exhibited a marked decrease in its value after exceeding 2 wt %. In turn, the deterioration of the binding and microstructural integrity of composites with high loadings of CB was due to the excessive specific surface area (254 m^2^ g^−1^) of this filler, which could not be properly wetted by insufficient amounts of the epoxy resin. In the case of GN filler, this problem was observed only at higher contents (6 wt %) than for CB. This was related to the significantly lower surface area of the GN used as compared to CB.

Summarizing, it is worth mention that obtained values of electrical conductivities from 178 to 267 S cm^−1^ depending on the exact composition, significantly exceeds the value of conductivity recommended by DOE (*σ* > 100 S cm^−1^) for materials for bipolar plates. Such good conductivity enables achieving low ohmic losses resulting from the interconnectors in PEMFC stack and is undoubtedly very beneficial for such type of devices.

### 3.4. Thermal Conductivity

**Figure 5:**
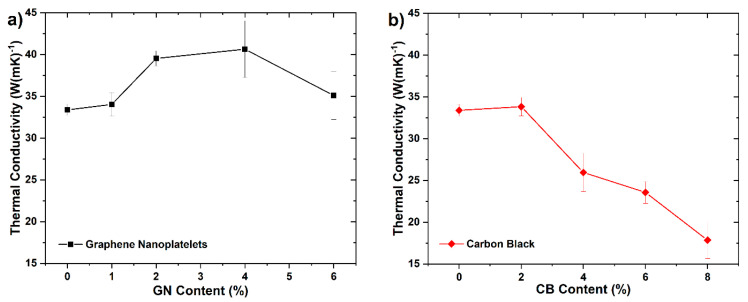
Thermal conductivity of composites depending on the content of secondary nanofillers: (**a**) graphene nanoplatelets, (**b**) carbon black.

The RT thermal conductivity of samples of composites measured using the LFA technique are shown in Figure 5. Investigation showed that addition of GN caused an increase in thermal conductivity of composites from about 33.4 to about 40.6 W mK^−1^ up to 4 wt % and after exceeding this content, it decreased to about 35 W mK^−1^. In the case of carbon black, we noted an initial slight increase in conductivity for the sample with 2 wt %, and then its value decreased monotonically to about 17.9 W mK^−1^. Nonetheless, even those less thermally conductive composites easily meet DOE targets for bipolar plates, which state that λ should be higher than 10 W mK^−1^ [33].

In order to gain more insight into the causes of the observed tendencies, we considered the contributions of the different components of heat transfer. It is well-known that the overall thermal conductivity of solids originates from the three basic modes of heat transfer—electronic transport, phononic transport and radiative transfer, whereas the two first modes are dominant in the low temperature regime. The first of these modes—electron conduction component may be estimated through the first-order approximation using Wiedemann–Franz law (Equation (1) [34]:

(1) $$\lambda_{e}=\sigma T\frac{\pi^{2}}{3}{\left(k_{B}/e\right)}^{2}$$

where *σ* is electric conductivity, *T* is absolute temperature, *k_B_* is the Boltzmann constant and *e* is the elementary electric charge. The calculation of the electronic transfer contribution in the composites with both nanofillers revealed that its values were in the range 0.13–0.19 W mK^−1^, which in most cases is far below 1% of the registered total thermal conductivity of composites. This indicates that *λ*, and its variations with added nanofillers predominantly resulted from the phonon transfer contribution, especially from the capacitive component and also from the Umklapp scattering due to the phonon-phonon interactions, which, nonetheless, should be relatively weak in the low temperatures [35,36]. Summarizing the above, the variation of the thermal conductivity of composites resulted mainly from the changes in their densities (related with specific heat capacity), stiffness, and overall binding of composites rather than electrical conductivity. In turn, the stiffness and microstructural integrity of materials influences the effectiveness of phonon transport and their scattering on the microstructural discontinuities and grain boundaries which is related to the interfacial thermal resistance. The correlation described is especially clear for CB composites, where the thermal conductivity dependence is almost the same as it is for apparent density and stiffness modulus-CB percentage plots. Another reason for the pronounced decline of thermal conductivity of composites with CB as contrary to the samples with GN, may be the strongly disordered structure of CB, which probably induced strong phonon scattering on its internal defects [37].

### 3.5. Flexural Properties

**Figure 6:**
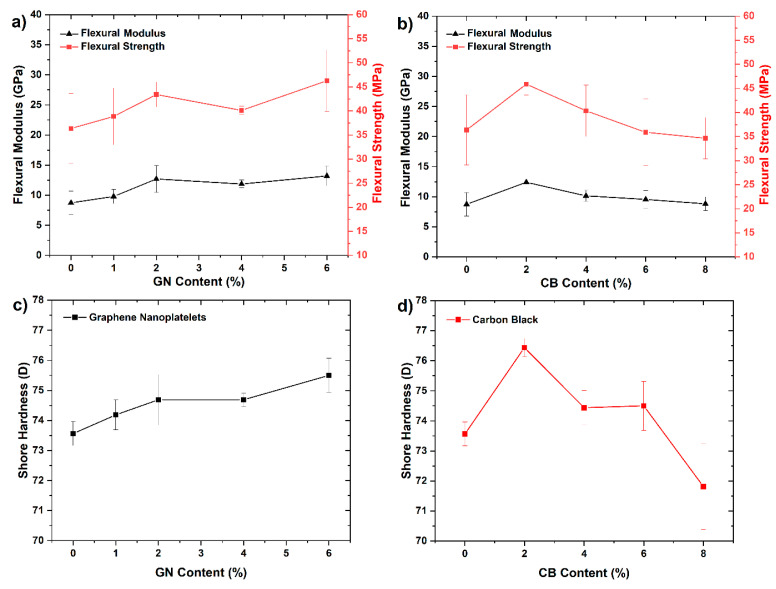
Flexural strength and modulus of EP/SG composites depending on the content of secondary nanofillers. (**a**) Graphene nanoplatelets; (**b**) carbon black; Shore hardness versus GN (**c**) and CB (**d**) percentage.

The mechanical properties are especially important, considering the potential application of investigated composites as interconnectors for PEMFC and similar devices. It is so because the bipolar plates must ensure proper stiffness, strength, and integrity of the cell stack which need to be tightly assembled as one. The changes in the flexural modulus, flexural strength, and shore hardness (SH) of EP/SG composites as a function of the secondary fillers content (GN and CB) in epoxy matrix are shown in Figure 6.

As shown in Figure 6, the conductive fillers introduced into the EP matrix exerted different but noticeable effects on the mechanical behaviors of the EP/SG samples. It is worth noting that the observed path of change in the mechanical properties of samples with filler content have a similar trend to the changes in the corresponding densities of composites (Figure 4). The greatest increase in flexural strength and modulus of EP/SG composites was achieved for 6 wt % of GN or 2 wt % of CB particles. The maximum increase in the flexural strength of EP/SG composites was 26% when 6 wt % of GN or 2 wt % of CB was added. Thus, an increase in modulus of composites by 51% at 6 wt % of GN and 42% at 2 wt % of CB was also demonstrated.

The literature review indicates various strengthening mechanisms of polymer composites by nanoparticles such as dispersion strengthening, packing density improvement and/or enhancing the interaction of the filler with the polymer matrix [31]. It is worth noting that due to the significantly larger SSA of CB as compared to GN (Table 1) the same maximum strength ~46 MPa can be achieved with a lower content of CB (2 wt %) than for GNs (6 wt %). As shown in Figure 6b, an increase in CB beyond 2 wt % significantly reduced the flexural strength and modulus of composite plates. This behavior can be explained by an inadequate amount of epoxy resin to wet and bind the carbon fillers, leading to the loss of structural integrity of the composites [38].

Contrary to CB filler, the use of graphene nanoplatelets resulted in a progressive growth in strength and modulus in line with an increase in GN content. The flexural and modulus curves shown in Figure 6a are very similar in variation to the composite density curves observed in Figure 4. Based on this correlation, it can be presumed that the increase in the strength and modulus of these composites takes place mainly due to the improvement in packing density of the fillers’ grains.

### 3.6. Shore Hardness

Loading of secondary fillers also changed the hardness of composites. As shown in Figure 6c, the addition of GN caused a gradual increase in the hardness of composites from 73.6 SH at 0 wt % to 75.5 SH at 6 wt % of GN. Such behavior can be attributed to filling the interstices between the larger graphite grains, causing the improvement in hardness accompanied by an increase in the apparent density of the composites. Because the GN used has a relatively low SSA, the majority of it was required to completely fill the interstices, so a continuous increase of hardness was observed.

EP/SG composites loaded with CB exhibited somewhat different behavior. Figure 6d shows that the addition of CB in the amount of 2 wt % caused an increase in hardness from 73.6 SH at 0 wt % to 76.4 SH at 2 wt % of CB; however, further addition led to a sharp drop in the hardness to 71.8 SH at 8 wt % of CB. As in the case of GN, the enhancement of hardness of composites with CB was mainly due to the packing density improvement—that is proved by its direct correlation with the apparent density of composites. The maximum packing density was already reached at 2 wt %, which is mainly due to the large SSA of CB. However, exceeding the amount of 2 wt % led to a reduction in hardness due to the poor wetting of CB by the resin, and consequently a poor overall bonding of these composites [31].

### 3.7. Thermal Stability

**Figure 7:**
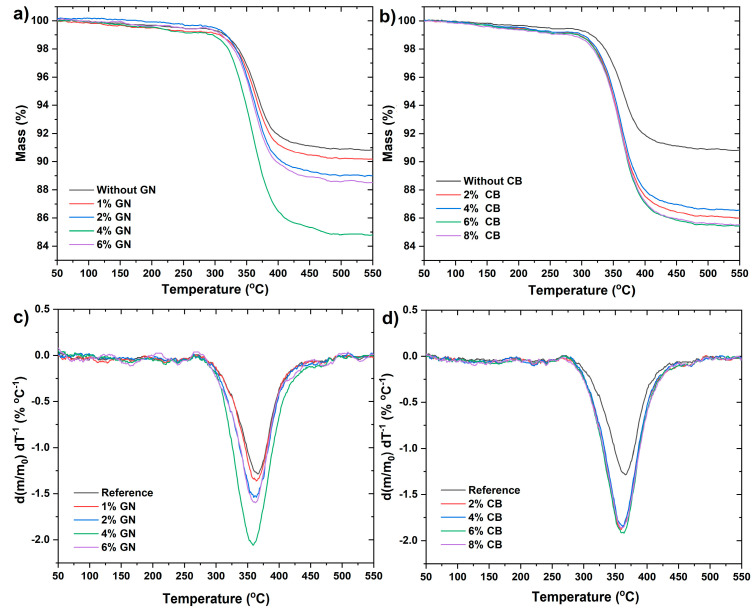
TG and DTG curves of EP/SG composites containing graphene nanoplatelets (**a**,**c**) and carbon black (**b**,**d**).

Thermogravimetric curves which demonstrate the thermal decomposition behavior of epoxy resin composites in a nitrogen atmosphere are shown in Figure 7a,b. The first graph shows the change in mass as a function of temperature for samples of composites based on resin and synthetic graphite modified with a different percentage of graphene nanoplates (Figure 7a), whereas Figure 7b presents the same relationship for epoxy-based composites but modified with carbon black. It can be seen that the thermal decomposition process of composite systems based on both GN and CB in a nitrogen atmosphere is similar to that of the unmodified epoxy resin; namely, it is a single step decomposition process. The differential thermogravimetry curve (DTG) also indicates a single step decomposition process of nanocomposites (Figure 7c,d).

**Figure 8:**
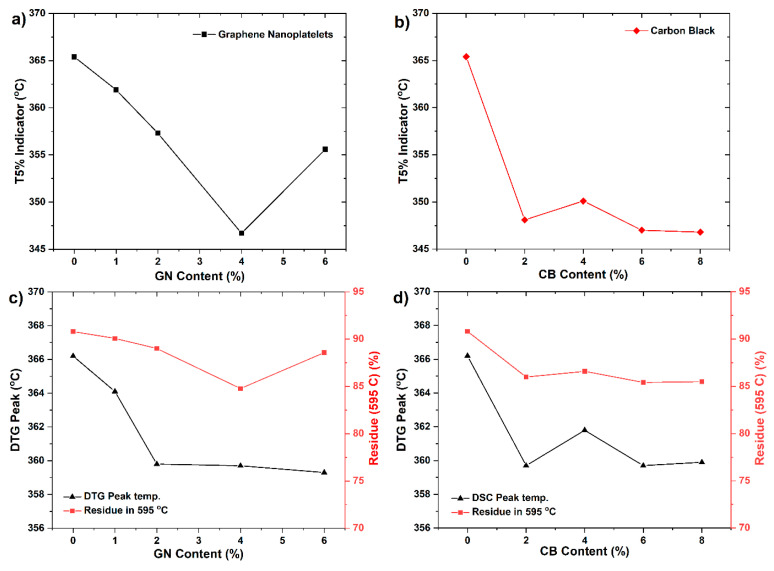
T5% indicator of composites with GN (**a**) and CB (**b**); decomposition peak and solid residue at 595 °C of composites with GN (**c**) and CB (**d**).

In general, when mass loss reaches up to 5% of the total weight of a sample, the corresponding temperature is defined as the starting decomposition temperature (T5%) of composites (Figure 8a,b). The presence of SG in epoxy composites increased the value of T5% thermal stability indicator in comparison with pure epoxy resin to about 140 °C (the results were not shown). In turn, the addition of GN and CB decreased the initial decomposition temperature in line with increasing nanoadditive content. For composites containing GN, the decrease in thermal stability is gradual, reaching the lowest value for 4 wt % of nanoadditive (Figure 8a). Whereas for composites containing CB, the decrease in stability is rapid, reaching the thermal stability value for 2 wt % of the nanoadditive (Figure 8b). Interestingly, further addition of CB no longer caused a decrease in thermal stability of composites—T5% value for 4%, 6%, and 8% of CB remains at the same level as for 2 wt % (Figure 8b).

The reason for such behavior may be the high SSA of CB particles, which is 254 m^2^ g^−1^, and is almost three times higher than for GN. High SSA is associated with the existence of a large concentration of active sites and functional groups capable of interacting with the polymer macromolecules [39], causing a deterioration in the wetting and binding of particles by the epoxy resin, which contributes to a decrease in the thermal stability of nanocomposites as well as a decrease in mechanical properties due to the increase in porosity. At the same time, the increase in percentage of CB with a high SSA probably causes the formation of agglomerates, which also contribute to the observed decrease in the thermal stability of composites [31,40].

As mentioned, the decrease in the thermal stability of nanocomposites with GN is more gradual than in nanocomposites with CB (Figure 8a,b), reaching the lowest value for 4 wt % of nanoadditive. While the further addition of modifying phases (6 wt %) increases the thermal stability in relation to 4 wt % of GN. The decrease in the temperature of 5% weight loss may be caused by the increase in the thermal conductivity of the polymer as an effect of the addition of GNs. Graphene nanoplates similarly to carbon nanotubes increase the heat diffusion which results in faster degradation of polymer. The results of our research indicate an increase in the thermal conductivity of nanocomposites with an increase in the weight fraction of GN, especially up to 4 wt % of the nanoadditive (Figure 5). The same tendency was reported in the literature [31,39,41]. The secondary increase in the thermal stability of samples with 6 %GN compared to 4 %GN samples may be due to the barrier effect of these nanoparticles, which are characterized by a high length-to-diameter ratio and homogeneous distribution of this nanoparticle in the matrix. The barrier effect of platelet-shaped nanoparticles such as montmorillonite or graphene, which block or delay the transfer of decomposition products from the condensed phase in composites to the surface or to the gas phase, is recorded in the literature [42,43,44]. The thermostability of polymers clearly depends on the degree of intercalation/exfoliation as in this case, of graphene layers—the better the dispersion of nanofiller which is achieved, the more significant the enhancement of thermal resistance can be expected. It is likely that the appropriate weight fraction combined with good dispersion, as well as the appropriate length to diameter ratio may affect the thermal stability of the composite.

The mass residue at 595 °C as a function of the amount of the modifying phase is strictly correlated with the thermal stability and the degradation temperature of these nanocomposites (Figure 8c,d). For the reference sample, the weight loss was 90.8%, while in the case of nanocomposites with GN, the greatest weight loss was observed for the 4% GN (84%), and the lowest for 1% GN (90.1%). On the other hand, for CB nanocomposites, the highest decrease was recorded for 2% CB (85.9%), while no significant differences were observed in the remaining CB samples when compared to the 2% CB sample.

Taking into account the potential use of these composites, e.g., as bipolar plates, their stability at temperatures up to 200–250 °C is very important. As was demonstrated in the performed tests, all of the investigated composites exhibit good stability up to mentioned temperature range, thus preserving the condition of thermal stability for bipolar plates for low- and medium-temperature fuel cells. Moreover, the addition of nanoparticles significantly enhanced electrical and mechanical properties of these composites.

## 4. Conclusions

In this work we present the results of the study on the effect of secondary carbon nanofillers on the electrical, thermal, and mechanical properties of conductive composites based on the high loadings of graphite and epoxy resin. The nanofillers chosen for examination were graphene nanoplatelets, which up to the present have rather rarely, and carbon black nanoparticles, which are the most frequently used secondary nanofiller, constituting the reference samples for comparison of the obtained data. The main purpose of such a comparison was to assess the potential of low-cost graphene nanoplatelets as an alternative secondary conductive nanofiller for CPC, aiming at improving electrical, thermal and mechanical performance of composites.

The performed tests showed that the addition of secondary nanofillers exerted a significant impact on the overall characteristics of composites; nonetheless, the extent of enhancements, the range of the proper contents of additives, and the causes of observed behaviors were different in the case of each nanofiller. In general, the composites with GN showed notably better properties as compared to those with CB, however, in order to achieve such improvements, larger contents of GN were needed as compared to CB. This behavior was related mainly to the significantly lower surface area of the GN used (SSA = 80 m^2^ g^−1^) than in the case of CB (SSA = 254 m^2^ g^−1^), and to its highly ordered and crystalline structure ensuring excellent electrical transport properties. By adding 4% of GN it was possible to achieve electrical conductivity of 266.7 S cm^−1^ (exceeding by 2.6 times the DOE requirements—DOER), thermal conductivity of 40.6 W mK^−1^ (4.0 times DOER), flexural strength of 40.1 MPa (1.6 times DOER), thermal stability (T5%) of 346.7 °C at the apparent density of 1.93 g cm^−3^. The observed enhancement was mainly due to higher packing density of fillers and high conductivity of graphene nanoplatelets, which together ensured reduced contact resistance between the individual conductive grains and lowered the porosity of the composites. In turn, the addition of small amounts of carbon black (~ 2 wt %) resulted in an improvement in mechanical properties of samples, accompanied by a decrease in their electrical and thermal conductivities. Moreover, the higher contents of CB caused a deterioration in the electrical, thermal, and, to a lesser extent, mechanical properties, mainly due to the insufficient amount of binder—epoxy resin, which could not properly wet and bind the fillers. This had a detrimental effect on the microstructural integrity of materials which caused a decline in the apparent density and other properties.

This work clearly shows the utility of low-cost graphene nanoplatelets as a secondary conductive nanofiller for CPC. The experiments performed also demonstrate the importance of other parameters of conductive fillers besides their conductivity, such as specific surface area, grain size, shape, and aspect ratio, which all exert a strong effect on the final properties of the composites. As revealed in the course of the performed investigation, the composites obtained show remarkable potential as materials for bipolar plates in low- and medium- temperature fuel cells but also sensors, antistatic materials, conductors resistant to corrosion, etc. As shown, the CPCs developed exceeds the majority of the most important parameters established by the US DOE, nevertheless, more extensive tests need to be performed such as H_2_ permeability, corrosion resistance, and operating device testing to fully assess the suitability of these materials for use in bipolar plates.

## Figures and Tables

**Table 1:** Morphological parameters of the carbon fillers used.

Property	Synthetic Graphite	Graphene Nanoplatelets	Carbon Black
Labelling	SG	GN	CB
Shape	Irregular, platelet	Platelet	Spheroidal
Particle sizes [µm]	10–200	1.5–10	0.03
Surface area (BET) [m^2^ g^−1^]	1.94	80	254

## Data Availability

The data presented in this study are available on request from the corresponding author. At the time the project was carried out, there was no obligation to make the data publicly available.
